# Game theory and environmental health behavior: a population model of sunscreen use, social norms, and melanoma incidence

**DOI:** 10.3389/fpubh.2026.1813270

**Published:** 2026-04-22

**Authors:** Miryam Kerner

**Affiliations:** 1 The Ruth and Bruce Rapoport Faculty of Medicine, Technion - Israel Institute of Technology, Haifa, Israel; 2 Kaplan Medical Center, Rehovot, Israel

**Keywords:** cost-effectiveness, economic burden, health economics, melanoma prevention, public health policy, sunscreen subsidies

## Abstract

**Introduction:**

Melanoma is a largely preventable yet economically significant cancer, particularly in high-UV regions. Despite strong evidence that sunscreen reduces melanoma risk, its use remains suboptimal due to misaligned private and social incentives.

**Methods:**

We develop an evolutionary game-theoretic model in which individuals choose between sunscreen use and non-use. Payoffs incorporate private benefits, costs, and externalities from shared healthcare financing. The model is calibrated using Australian data.

**Results:**

Behavior is driven by private incentives. When private benefits are lower than costs, populations converge to non-adoption, even when sunscreen use is socially optimal. Calibration shows this divergence arises in high-risk populations.

**Discussion:**

Sunscreen use represents a public goods problem with under-adoption driven by externalities. Policies such as subsidies, mandates, and social norm interventions are required to align incentives and improve public health outcomes.

## 1 Introduction

Melanoma is one of the most preventable cancers globally, yet its economic burden remains substantial. Countries with high ultraviolet (UV) exposure, particularly Australia and New Zealand, exhibit some of the highest lifetime risks of melanoma. Randomized and longitudinal evidence demonstrates that regular sunscreen use can significantly reduce melanoma incidence (1). However, despite this well-established clinical effectiveness, adherence to recommended sunscreen use remains inconsistent and often suboptimal across populations (2, 3).

This gap between proven effectiveness and real-world behavior presents an important economic and public health puzzle. In particular, individuals may undervalue preventive actions whose benefits are partly realized at the societal level (4, 5). While sunscreen use provides direct private benefits through reduced individual melanoma risk, it also generates broader social benefits by lowering the expected burden on publicly financed or pooled healthcare systems (6, 7).

This divergence between private incentives and collective welfare suggests that sunscreen use may be usefully conceptualized as a public goods problem. Preventive health behaviors often exhibit features of positive externalities and partial non-excludability, particularly in systems with shared healthcare financing, meaning that individual preventive actions generate benefits that extend beyond the individual to the broader population (8). Individuals who choose not to use sunscreen do not fully internalize the social costs associated with higher expected treatment expenditures, leading to underinvestment in prevention relative to the social optimum. In addition, behavioral biases such as present bias, limited risk perception, and imperfect information may further contribute to under-adoption of preventive measures (9, 10), reinforcing the gap between individually optimal and socially optimal behavior.

In this context, the free-rider problem becomes particularly salient. Individuals may rationally choose to avoid the private costs of sunscreen use—such as monetary expense or inconvenience—while implicitly relying on the healthcare system to absorb potential future treatment costs. This mechanism creates a form of cost externalization; whereby individual decisions generate spillover effects that are not fully reflected in private decision-making.

To analyze these dynamics, this paper applies an evolutionary game-theoretic framework to model sunscreen adoption as a strategic interaction between individuals. Specifically, individuals choose between cooperation (using sunscreen) and defection (not using sunscreen), with payoffs determined by private benefits, private costs, and external effects arising from shared healthcare financing. Unlike standard coordination problems, the model highlights how misaligned incentives—rather than strategic uncertainty—can drive socially inefficient outcomes.

An evolutionary game-theoretic framework is particularly appropriate in this context because sunscreen use represents a repeated behavioral decision that evolves over time at the population level. Individuals are not assumed to solve fully rational, forward-looking optimization problems; rather, behavior adapts through experience, social influence, and observed outcomes. Unlike standard game-theoretic models based on static equilibrium concepts, the evolutionary approach captures how preventive behavior emerges and stabilizes under decentralized decision-making and externalities. In this framework, individuals do not condition their behavior on the past actions of specific others (as in repeated or tit-for-tat interactions) but instead respond to aggregate outcomes and incentives at the population level. This is especially relevant in public health settings, where compliance is shaped by both individual incentives and population-level dynamics.

The presence of positive externalities and free-riding in sunscreen use provides a clear rationale for policy intervention. Governments may seek to correct this market failure through instruments such as subsidies for sunscreen products, public information campaigns, or regulatory approaches such as mandatory provision in high-exposure environments (e.g., schools, workplaces, and beaches), particularly in regions with high UV exposure where melanoma incidence is elevated. From a welfare perspective, such interventions can be interpreted as mechanisms to internalize external costs and align private incentives with social optimality (11). Moreover, policies that leverage social norms or behavioral nudges may complement traditional economic instruments by addressing non-monetary barriers to preventive behavior. Understanding sunscreen use through a public goods framework thus offers a structured basis for evaluating the efficiency and design of preventive health policies.

Building on prior work applying game-theoretic frameworks to health-related behavior (12, 13), the present study extends this approach by modeling sunscreen use as a public goods problem driven by indirect externalities arising from shared healthcare financing. By framing sunscreen use as a public goods problem, the paper contributes to the literature on health behavior and preventive care by providing a formal mechanism through which under-adoption can emerge, even when prevention is socially efficient. The model further offers clear policy implications, demonstrating how interventions such as subsidies, mandates, or social norm mechanisms can shift behavior toward socially optimal levels of adoption.

## 2 Literature review

Public goods theory, first formalized by Samuelson (14), describes goods that are non-rival and non-excludable. Sunscreen use exhibits these characteristics, where one individual’s use of sunscreen reduces the societal costs of melanoma treatment without excluding others from benefiting. However, as Olson (15) points out, individuals often avoid contributing to the provision of public goods if they can benefit from others’ contributions, resulting in the free-rider problem.

Sunscreen use fits into the category of a quasi-public good, where the benefits are both private (personal melanoma risk reduction) and public (reduced overall melanoma treatment costs). However, individuals are unlikely to fully internalize the social benefits, leading to suboptimal sunscreen adoption. Several public health campaigns and policy measures have tried to address this issue by promoting increased sunscreen use, yet these efforts have not always achieved the desired outcome due to the free-rider problem.

Game-theoretic approaches have been widely used to model health-related behavior, particularly in the context of vaccination and compliance dynamics (16) and more broadly in pandemic-related behavioral responses (12, 13). This paper builds on these models, applying evolutionary game theory to study sunscreen adoption. In the proposed model, individuals choose between cooperation (using sunscreen) and defection (not using sunscreen), with the payoff matrix accounting for both personal and external benefits.

## 3 Theoretical model

To analyze the dynamics of sunscreen adoption, I adopt an evolutionary game-theoretic framework. This approach is particularly suitable in the present context because sunscreen use represents a repeated behavioral decision that evolves over time at the population level, rather than a one-time strategic choice. Individuals are not assumed to solve fully rational optimization problems; instead, behavior adjusts through adaptation, social influence, and observed outcomes. This allows the model to capture how preventive behavior emerges and stabilizes under decentralized decision-making and externalities, which are central features of public health settings.

The model considers a population in which individuals choose between cooperation (using sunscreen) and defection (not using sunscreen). The key features of the model include private benefits, external costs, and private costs associated with sunscreen use.

Each individual in the population must decide whether or not to use sunscreen. The key feature of this model is that sunscreen use reduces an individual’s probability of developing melanoma. In the presence of publicly funded or pooled health systems, melanoma treatment costs are partly borne by society. Therefore, individual decisions generate spillover effects through the sharing of treatment costs.

### 3.1 Payoff structure

Each individual has a payoff based on their decision (whether or not to use sunscreen) and the strategies of others in the population.

The personal benefit of cooperation results from reduced melanoma risk, and potentially social influences (not modeled explicitly in the baseline framework). When people observe others using sunscreen, they may be more likely to follow this behavior in order to conform to prevailing norms.

#### 3.1.1 For Cooperators (*C*)

- *Personal Benefit (B)*: Cooperators reduce their own risk of melanoma by using sunscreen.
- *Cost (C)*: there is a private cost associated with using sunscreen, such as money or time spent on applying it.
- *External Effect (Positive Spillover)*: by reducing their own probability of illness, cooperators lower the expected burden on the healthcare system and thereby reduce costs that would otherwise be shared by others.

#### 3.1.2 For Defectors (*D*)

- *No Personal Benefit*: Defectors do not use sunscreen and therefore do not reduce their own melanoma risk.
- *No Cost*: Defectors avoid the private cost of sunscreen use.
- *External Cost (E) (Negative Spillover)*: because melanoma treatment is partly publicly financed, defectors impose an expected cost on others by increasing the likelihood of future treatment expenditures.

### 3.2 Payoff matrix

The payoff matrix captures the interactions between two individuals. I incorporate the negative spillover arising from publicly shared melanoma treatment into the payoff structure (see Table 1).

- *Cooperator vs. Cooperator (C vs C)*: When two cooperators interact, both reduce their melanoma risk and incur the cost of sunscreen. Since neither imposes additional burden on the healthcare system, both receive the net private payoff B−C .
- *Cooperator vs. Defector (C vs D)*: the cooperator reduces their own risk and pays the cost C , but is exposed to the external cost generated by the defector through the public financing of treatment. The cooperator’s payoff is therefore B−C−E . The defector avoids the cost of sunscreen and receives no direct benefit, yielding a payoff of 0 .
- *Defector vs. Cooperator (D vs C)*: this is symmetric to the previous case. The defector avoids the cost of sunscreen and receives no direct benefit, while the cooperator bears the external cost associated with the defector’s behavior.
- *Defector vs. Defector (D vs D)*: When both defect, neither incurs the cost of sunscreen, but both contribute to higher expected melanoma treatment costs that are shared through the public system. Each therefore bears the external cost E , resulting in a payoff of −E .

**Table 1:** Payoff matrix.

	Cooperator (*C*)	Defector (*D*)
Cooperator (*C*)	(B−C),(B−C)	(B−C−E),0
Defector (*D*)	0,(B−C−E)	(−E,−E)

### 3.3 Social dynamics and public goods dilemma

This setup generates a social dilemma analogous to a public goods problem. However, the mechanism differs from standard settings in which cooperation directly benefits others. In the present model, the externality arises through the financing of melanoma treatment in a public or pooled healthcare system.

Individuals who do not use sunscreen increase their probability of requiring treatment, and part of the associated cost is borne by others. As a result, individuals do not fully internalize the social cost of non-use. This creates a form of cost externalization, whereby defectors avoid the private cost of prevention while shifting part of the expected burden onto society.

Consequently, sunscreen use may be lower than the socially optimal level, even though widespread cooperation would reduce the overall economic burden of melanoma.

In this context, the externality takes the form of a negative fiscal spillover, whereby individuals who do not adopt preventive behavior impose costs on others through shared healthcare financing.

### 3.4 Evolutionary dynamics

To analyze how sunscreen adoption evolves over time, I model the population using standard replicator dynamics. Let x∈[0,1] denote the proportion of cooperators (individuals who use sunscreen), and 1−x the proportion of defectors.

Based on the payoff structure defined above, the expected payoffs are:


$$\pi_{C}=B-C-E(1-x)$$

$$\pi_{D}=-E(1-x)$$

The intuition is straightforward: both strategies are exposed to the negative externality generated by defectors through shared healthcare costs, but only cooperators incur the private cost C and receive the private benefit B .

The average payoff in the population is given by:


$$\bar{\pi }=x\pi_{C}+(1-x)\pi_{D}$$

The evolution of cooperation is governed by the replicator equation:


$$\dot{x}=x(\pi_{C}-\bar{\pi })$$

Substituting and simplifying yields:


$$\dot{x}=x(1-x)(B-C)$$

This expression fully characterizes the evolutionary dynamics of sunscreen adoption.

Importantly, because both the private benefit B and private cost C are independent of the population share of cooperators, the payoff difference between strategies does not depend on population composition. As a result, the evolutionary dynamics are driven solely by private incentives.

### 3.5 Equilibrium and stability analysis

Equilibria occur when $\dot{x}=0$ , which gives:


$$x^{*}=0\;\text{and}\;x^{*}=1$$

The direction of selection depends entirely on the sign of (BC) .

**Figure 1:**
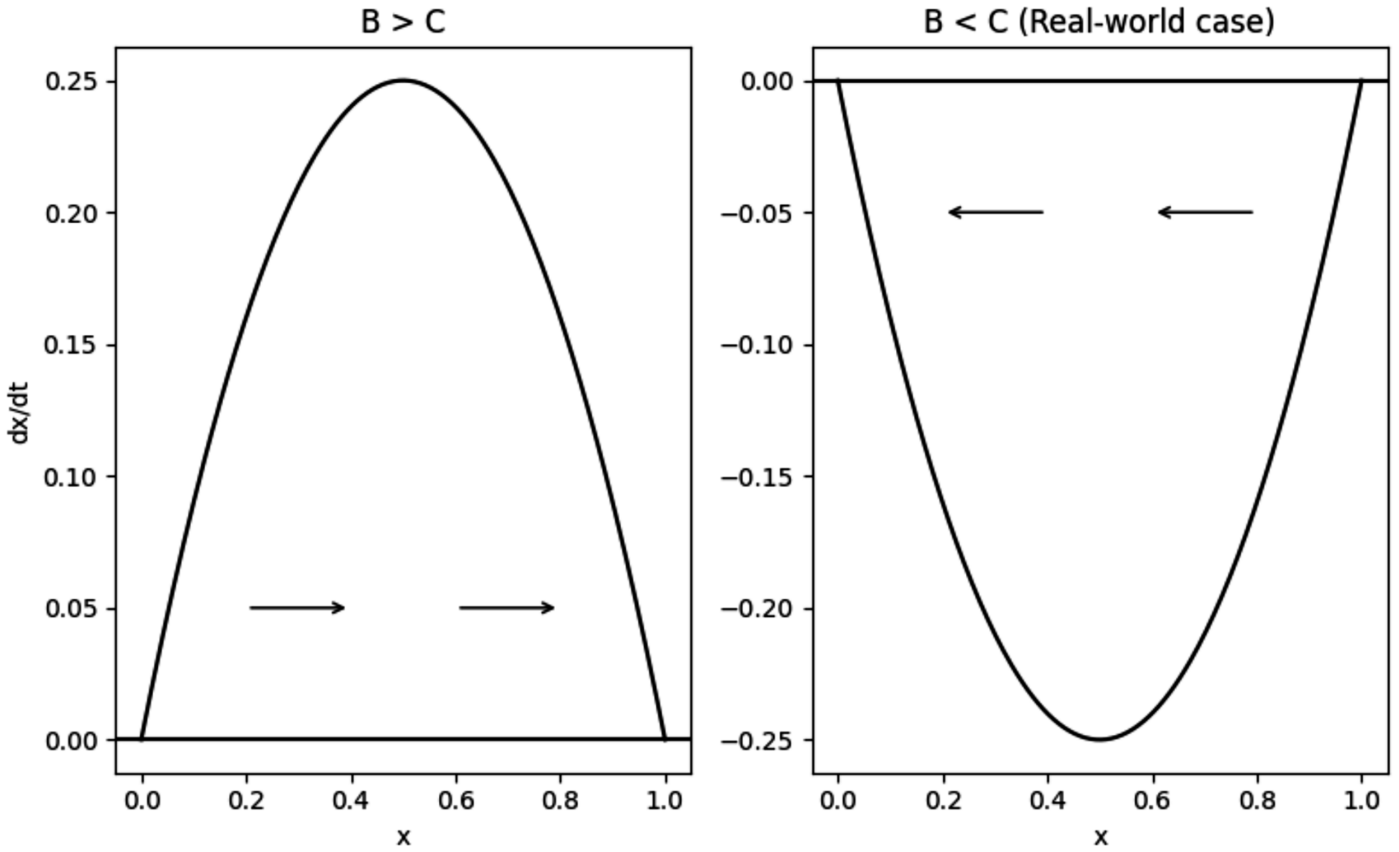
Evolutionary dynamics of sunscreen adoption.

Figure 1 illustrates these dynamics. The horizontal axis represents the share of cooperators x , while the vertical axis shows the growth rate $\dot{x}$ . Arrows indicate the direction of evolutionary change.

#### 3.5.1 Case 1: *B > C* (private incentives aligned)

As shown in Panel (a) of Figure 1, the growth rate $\dot{x}$ is positive for all interior values of x . The system therefore moves monotonically toward full cooperation. In this case, the equilibrium x=1 is globally stable, while x=0 is unstable.

#### 3.5.2 Case 2: *B < C* (free-riding regime)

Panel (b) of Figure 1 depicts the empirically relevant case. Here, the growth rate $\dot{x}$ is negative for all interior values of x , implying that cooperation declines over time. The equilibrium x=0 is globally stable, while x=1 is unstable. The population converges to complete non-adoption of sunscreen.

### 3.6 Interpretation: externalities without strategic feedback

A central feature of this model is that the externality E cancels out in the evolutionary dynamics. Although defectors impose costs on society through publicly shared treatment expenditures, these costs affect both strategies symmetrically and therefore do not influence the direction of selection.

As a result, behavior is determined solely by private incentives. This produces a sharp divergence between:

- *Evolutionary stability*, determined by B−C , and
- *Social optimality*, determined by B+E−C

This divergence generates a classic public goods problem:

- Even when B+E>C (cooperation is socially efficient),
- If B<C , individuals will rationally defect.

Figure 1 highlights that, in the absence of intervention, the system converges to the boundary equilibrium dictated by private incentives.

An important implication of this structure is that the absence of strategic feedback arises from the assumption that private benefits and costs are independent of population behavior. In the present setting, the externality operates symmetrically through shared costs and therefore does not affect the relative payoff between strategies. As a result, behavior is determined solely by private incentives, and the model does not generate interior equilibria or tipping points but instead exhibits a dominance structure in which one strategy is preferred regardless of the behavior of others. In extensions where either the perceived benefits of sunscreen use or its costs depend on the prevalence of adoption—for example, through social norms, peer effects, or behavioral spillovers—the payoff difference may become a function of the population state, potentially giving rise to interior equilibria or tipping points.

This result has direct policy implications. Since the inefficient equilibrium arises from misaligned private incentives rather than coordination failure, informational campaigns alone are unlikely to be sufficient. Effective interventions must directly alter the payoff structure—through subsidies, mandates, or cost-sharing mechanisms—to shift the system from the B<C regime to the B>C regime.

### 3.7 Policy intervention and incentive alignment

Policy interventions can be interpreted in the model as mechanisms that modify the private payoff structure. In particular, a subsidy s that reduces the private cost of sunscreen use transforms the payoff of cooperators to:


$$\pi_{C}=B-(C-s)-E(1-x)$$

The replicator dynamic then becomes:


$$\dot{x}=x(1-x)(B-C+s)$$

This implies that policy can shift the system from the free-riding regime ( B<C ) to the cooperation regime ( B>C ) whenever:


s>C−B

Thus, even when sunscreen use is privately unattractive, sufficiently strong subsidies can restore full cooperation as the globally stable equilibrium.

More broadly, any intervention that increases private benefits (e.g., social incentives, workplace enforcement) or reduces private costs (e.g., subsidies, free provision) operates by shifting the evolutionary dynamics across regimes. The phase diagram therefore provides a simple criterion for policy effectiveness: interventions must be large enough to change the sign of B−C .

## 4 Calibration for Australia

We calibrate the model using data from Australia, focusing on lifetime melanoma risk, treatment costs, and sunscreen costs. Australia is known for high UV radiation and melanoma risk. However, considering the presence of susceptible populations in other countries, this model is also applicable to nations like the United States.

The following scenario assumptions and parameters are used:

### 4.1 Scenario assumptions and parameters

- Exposure: *90 sunny days per year*
- Sunscreen quantity: *20 mL/day* (moderate “exposed skin” use)
- Horizon: *60 summers* (roughly ages 15–75)
- Private cost share: *θ = 0.20* (illustrative for mixed public/private + time costs)
- Risk reduction: *50%* (RR = 0.50). This value is based on follow-up data from a trial conducted by Green et al. (1) (see Table 2).

**Table 2:** Parameters (Australia).

Symbol	Meaning	Value used	Evidence/note
*p*	Lifetime melanoma risk	0.0674	AIHW lifetime incidence risk figure
RR	Relative risk with daily sunscreen	0.50	RCT follow-up HR ≈ 0.50
*K*_med	Direct medical cost per melanoma case	11,788 AUD	Derived from published totals & counts
*V*	AUD per QALY	75,000 AUD	*Assumption* (show sensitivity in paper)
ΔQALY	QALY loss per melanoma case	1.0	*Assumption* (can be stage-weighted)
*K*	Total per-case monetized loss	86,788 AUD	$K_{\mathrm{med}}+\text{VΔQALY}$
Price	Sunscreen price	10.99 AUD/110 mL	Chemist Warehouse
mL/day	Sunscreen use intensity	20 mL/day	*Assumption* (exposed areas)
Days	Sunny days per summer	90	*Scenario choice*
Years	Summers	60	*Scenario choice*
*θ*	Private share of *K*	0.20	*Assumption* (varies by system)

Notes:

1. Lifetime melanoma risk [AIHW (17)]: The paper uses the Australian Institute of Health and Welfare [AIHW (17)] lifetime melanoma risk figure, which is 0.0674. This is the lifetime probability of developing melanoma in Australia. This figure is sourced from AIHW (17), which compiles data on health statistics in Australia.
2. Relative risk reduction with daily sunscreen (1): the study cites a 50% relative risk reduction (RR = 0.50) based on randomized controlled trial follow-up data. Green et al. (1) conducted a study showing that regular sunscreen use reduces melanoma risk by 50%. This value was used in the model to calculate the impact of sunscreen use on individual melanoma risk.
3. Medical costs [AIHW (17)]: the direct medical cost per melanoma case is taken as 11,788 AUD, sourced from published totals and counts. This figure represents the average cost for medical treatment per individual diagnosed with melanoma in Australia. It’s an aggregate figure that includes treatments such as surgery, chemotherapy, and follow-up care.
4. Sunscreen price (18): the price for sunscreen is taken as 10.99 AUD per 110 mL, sourced from Chemist Warehouse (18). This is the retail price for sunscreen in Australia, which is used to estimate the personal cost of using sunscreen over a period of time.

Explaining the 86,788 AUD Figure:

The 86,788 AUD figure represents the total per-case monetized loss due to melanoma, including both medical treatment costs and the associated costs to society, such as lost quality-adjusted life years (QALYs). Here’s a breakdown of how this is derived:

1. Direct Medical Cost per Melanoma Case (*K*_med): The direct medical costs for treating melanoma, as cited from AIHW (17), is 11,788 AUD.
2. Value of a quality-adjusted life year (QALY): the paper assumes a value of 75,000 AUD per QALY (which is a standard valuation used in health economics to represent the monetary value of one year of perfect health).
3. QALY loss per melanoma case (ΔQALY): The QALY loss per melanoma case is assumed to be 1.0, which represents a full year of quality-adjusted life lost due to melanoma, or the loss in health-related quality of life due to the disease.
4. Total monetized loss (*K*): The total per-case monetized loss, 86,788 AUD, is calculated as the sum of the direct medical costs and the monetized loss due to the reduction in QALYs:

$$K=K_{\mathrm{med}}+(V\times \Delta \text{QALY})$$

K=11,788AUD+(75,000AUD/QALY×1.0)=86,788AUD

5 This figure represents the full economic burden of a melanoma case to society, including both medical treatment and the lost quality of life.

### 4.2 Computation of B, E, C and free-riding condition

First compute the lifetime sunscreen cost:

- Price per mL ≈ 10.99/110 = 0.0999 AUD/mL
- *C* = 20 × 0.0999 × 90 × 60 = 10,790 AUD

Now compute benefits:


B+E=p(1−RR)K=p·0.5·86,788

B=θ(B+E),E=(1−θ)(B+E)

### 4.3 Interpretation

Table 3 highlights how the relationship between private and social incentives varies across risk levels. For the average-risk case in Australia, both the private benefit B and the total social benefit B+E remain below the private cost C , implying that sunscreen use is neither privately nor socially justified under these parameters. Thus, the model suggests that the public goods problem is not universal but emerges only in sufficiently high-risk populations.

**Table 3:** Calibration of private incentives, social efficiency, and policy thresholds (Australia).

Population risk p	B+E (AUD)	B (AUD)	E (AUD)	C (AUD)	Private incentive	Social efficiency	Evolutionary outcome	Minimum subsidy $s^{*}=C-B$ (AUD)
0.067 (Australia average)	2,924	585	2,339	10,790	B<C	B+E<C	Non-adoption (efficient)	10,205
0.20 (high risk)	8,679	1,736	6,943	10,790	B<C	B+E<C	Non-adoption (efficient)	9,054
0.25 (very high risk)	10,849	2,170	8,679	10,790	B<C	B+E>C	Non-adoption despite social gains (inefficient)	8,620
0.30 (extreme risk)	13,018	2,604	10,415	10,790	B<C	B+E>C	Non-adoption despite social gains (inefficient)	8,186

In contrast, for sufficiently high-risk groups, a divergence emerges between private and social incentives. Specifically, while the total welfare gain B+E exceeds the cost C , the private benefit B remains below C . In these cases, sunscreen use is socially efficient but privately unattractive.

Under the revised evolutionary dynamics, this distinction has clear implications. Since behavior is determined by the sign of B−C , these high-risk populations remain in the B<C regime from the perspective of private incentives. As illustrated in Figure 1, this implies convergence toward zero adoption in the absence of intervention, despite the presence of substantial social benefits.

More generally, the results show that a divergence between private incentives and social efficiency emerges only beyond a threshold level of melanoma risk. In the present calibration, this threshold occurs at approximately p≳0.25 , where the condition B+E>C first holds while B<C continues to apply.

The calibration therefore identifies a parameter region in which laissez-faire behavior leads to inefficient outcomes. Individuals do not internalize the external benefit E , and as a result, preventive behavior is underprovided relative to the social optimum. This gap between evolutionary stability and social efficiency provides a clear economic rationale for policy intervention.

This result reflects expected-value calculations over a long horizon and does not contradict medical recommendations, which may account for risk aversion, heterogeneous exposure, and behavioral factors.

From a policy perspective, the model yields a simple threshold condition. As shown in Section 3.7, an intervention such as a subsidy s can shift the system from the non-adoption regime to full adoption whenever s>C−B . Under the calibration in Table 3, this implies that for high-risk populations the required subsidy is substantial. For example, when p=0.25 , the threshold is approximately s>8,620 AUD, while for p=0.30 it remains above s>8,186 AUD.

These magnitudes reflect the fact that, although the social benefits of sunscreen use are large, they are only weakly internalized at the individual level. The phase diagram can therefore be interpreted as a policy map: sufficiently strong interventions are required to shift the system from the B<C regime to the B>C regime, thereby restoring cooperation as the stable outcome.

### 4.4 Estimation of public costs for melanoma treatment

To better understand the potential public cost of melanoma treatment, a revised calculation is conducted using more realistic demographic parameters. This estimation assumes the following:

Assumptions:

- Total population: 27,600,000
- Proportion of population aged 60+: 23%
- Proportion of males among those aged 60+: 49.6%
- Proportion of population with higher melanoma risk (e.g., fair skin, European ancestry): 60% (adjusted from the unverified estimate of 75%)
- Estimated cost per melanoma case: AUD 8,679

Using these adjusted parameters, the estimated cost of melanoma treatment for the high-risk older Australian population is calculated as follows:


27,600,000×0.23×0.496×0.60×8,679≈18,367,558,099

This yields a rough estimate of AUD 18.4 billion in potential public expenditure on melanoma treatment for high-risk older individual males. This assumes that every person in the high-risk population will incur the cost of one melanoma treatment case.

So, if the estimated public cost of melanoma treatment for high-risk older Australians is AUD 18.4 billion, then in US Dollars this is roughly:


18.4billionAUD×0.71USD/AUD≈13.1billionUSD

Therefore, the corresponding estimate in US dollars is approximately USD 13.1 billion (based on the current exchange rate).

It is important to note that this calculation is based on a simplified model. The actual public costs of melanoma treatment would depend on a range of factors, including incidence rates, survival rates, the effectiveness of prevention interventions, and healthcare pricing.

#### 4.4.1 Key considerations for the estimates

1. Treatment costs per patient: the figure of USD 8,679 represents an estimated average treatment cost. Actual costs can vary depending on the type of care (e.g., surgery, immunotherapy, follow-up care) and the stage of melanoma at diagnosis. For more accurate and region-specific data, Australian melanoma treatment costs can be examined further.
2. Risk distribution: not all individuals in the high-risk group will develop melanoma. While the lifetime risk for melanoma is higher in those with fair skin or European ancestry, it remains significantly lower than 25%, even within this subgroup.
3. Age cutoff: the use of an age cutoff of 60+ (as opposed to 65+) expands the size of the older population cohort. This is beneficial for modeling the broader future healthcare burden, though melanoma risk notably peaks in older age groups, especially between the ages of 60 and 79.
4. Fair skin and ancestry proxies: given the lack of skin color data in Australian health statistics, ancestry (e.g., European descent) is used as a proxy for estimating the relative melanoma risk. This approach may either overestimate or underestimate the actual biological risk associated with melanoma.

## 5 Discussion

The results of this study show that sunscreen adoption can be understood as a public good problem arising from a divergence between private incentives and social welfare. While sunscreen use generates both private and social benefits, individuals do not fully internalize the external costs associated with melanoma treatment in publicly financed or pooled healthcare systems. As a result, preventive behavior may be underprovided relative to the social optimum.

This finding is consistent with the broader literature on public goods and health behavior. In particular, it aligns with the theoretical framework of Arrow (4) and Pauly (6), which emphasizes how health-related decisions are affected by externalities and insurance structures. More broadly, the results are consistent with public goods theory (14, 15), where individually rational behavior can lead to socially inefficient outcomes due to free-riding. The present study extends this literature by providing a formal mechanism through which such inefficiencies can arise in the context of preventive health behavior, even when the externality operates indirectly through shared healthcare financing rather than direct interpersonal effects.

The model also relates to prior work using game-theoretic approaches in public health, particularly in the context of vaccination and compliance behavior (16) and pandemic-related behavioral responses (12, 13). However, a key distinction in the present setting is that the externality does not generate strategic feedback between individuals. Instead, it operates symmetrically through shared costs, implying that behavioral dynamics are driven primarily by private incentives rather than coordination or expectation-based interactions. This result highlights an important difference between preventive behaviors such as sunscreen use and other health behaviors where social interactions generate tipping points or multiple equilibria.

The calibration results further refine this insight by showing that the public goods problem is not universal but emerges only beyond a threshold level of melanoma risk. For lower-risk populations, sunscreen use may be neither privately nor socially optimal under the assumed parameters. In contrast, for sufficiently high-risk groups, a divergence arises in which sunscreen use is socially efficient but privately unattractive. This finding is consistent with empirical evidence showing heterogeneous adoption of preventive behaviors across populations (2, 3), and it suggests that policy interventions should be targeted rather than uniform.

From a policy perspective, the results provide a clear and structured interpretation of intervention effectiveness. Because the inefficient equilibrium arises from misaligned private incentives rather than coordination failure, informational campaigns alone are unlikely to be sufficient. Instead, effective policies must directly modify the payoff structure faced by individuals. In the context of the model, this can be achieved through subsidies that reduce the private cost of sunscreen use, mandates in high-exposure environments, or interventions that increase perceived private benefits, such as social norm mechanisms. These findings are consistent with economic approaches to correcting externalities (11) and with recent work emphasizing the role of behavioral and social factors in shaping preventive health decisions (9, 10).

The model also provides a useful framework for interpreting the role of social norms. While the baseline formulation does not generate endogenous tipping points, the inclusion of social influence can alter the effective payoff structure and potentially support higher levels of cooperation. This is consistent with evidence from public health interventions showing that norm-based strategies can complement economic incentives in promoting behavior change.

More broadly, the framework developed in this paper has implications beyond melanoma prevention. Many environmental and public health behaviors—such as vaccination, occupational safety, and climate-related health risks—exhibit similar features, where private incentives are insufficient to generate socially optimal outcomes. By modeling these behaviors within an evolutionary game-theoretic framework, it is possible to capture how individual decisions aggregate into population-level outcomes and to identify the conditions under which policy intervention is required.

### 5.1 Limitations

This study has several limitations that should be acknowledged. First, the model assumes a homogeneous population, which abstracts from heterogeneity in risk exposure, preferences, and behavioral responses. In practice, individuals differ substantially in terms of melanoma risk, income, access to sunscreen, and responsiveness to policy interventions. Second, the calibration relies on stylized parameter values and simplifying assumptions, particularly with respect to QALY valuation and cost estimates, which may vary across settings. Third, the model is based on an expected-value framework and does not explicitly incorporate risk aversion or uncertainty in individual decision-making. Fourth, the evolutionary dynamics assume that individuals adapt behavior based on aggregate outcomes, without modeling detailed psychological or informational processes.

Future research could extend the model by incorporating heterogeneity across individuals, stochastic dynamics, and richer behavioral mechanisms. Empirical validation using observed data on sunscreen use and melanoma incidence would also be valuable in assessing the quantitative relevance of the model’s predictions.

## 6 Policy implications

To overcome the free-rider problem and promote sunscreen use, several policy interventions are recommended. First, sunscreen subsidies should be implemented, particularly for high-risk groups such as outdoor workers and individuals with fair skin, in order to reduce the cost of cooperation and incentivize greater adoption. Making sunscreen more accessible and affordable will be key to ensuring that high-risk populations are adequately protected.

Second, public health campaigns should focus on raising awareness about the societal benefits of sunscreen use, particularly in terms of reducing melanoma treatment costs for society. These campaigns should also promote behavior change by emphasizing the collective responsibility of sunscreen use for personal and public health.

Third, mandates for high-risk populations should be introduced, requiring sunscreen use in settings where individuals are at heightened risk, such as construction sites and agricultural fields. These mandates would not only protect individuals but also contribute to lowering overall melanoma rates, thus benefiting public health systems by reducing the financial burden of melanoma treatments.

Fourth, social norms should be fostered around sunscreen to shift individual behavior toward cooperation. By encouraging sunscreen as a social norm, especially in high-risk communities, more individuals would be motivated to adopt sunscreen use as part of their daily routine.

The inclusion of social norm dynamics in our model suggests that extensions incorporating social influence could generate multiple equilibria, highlighting the importance of peer influence and cultural expectations in shaping preventive behavior. Populations can self-organize around low-prevention equilibria, perpetuating higher disease incidence, or around high-prevention equilibria, where social reinforcement encourages widespread adoption of protective behaviors. This suggests that policy interventions leveraging social influence, such as public campaigns, peer education, and norm signaling, can shift behavior toward the socially optimal equilibrium.

Beyond melanoma, this framework has broader relevance to low-probability, high-cost environmental health risks, where private incentives alone are insufficient to achieve population-level prevention. Examples include behaviors affecting chronic diseases, occupational exposures, and climate-related health hazards. Incorporating economic, behavioral, and social factors into evolutionary game-theoretic models allows policymakers to anticipate potential equilibrium outcomes and design interventions that align individual and societal interests.

Finally, research and monitoring should be an ongoing effort to evaluate the effectiveness of sunscreen interventions, refine strategies, and maximize social welfare benefits. Future research should also investigate the psychological and social factors that influence sunscreen adoption and explore additional methods to increase compliance.

By combining these policies, it is possible to address the free-rider problem and significantly increase cooperation, leading to a substantial reduction in melanoma rates. Sunscreen use is a critical public health intervention, and the insights provided by this evolutionary model can guide future efforts to improve its adoption across populations.

## Data Availability

Publicly available datasets were analyzed in this study. This data can be found here: https://www.aihw.gov.au/.
